# Malignancies in AIDS patients: the experience of a tertiary hospital in a high prevalence zone

**DOI:** 10.1186/1750-9378-7-S1-P17

**Published:** 2012-04-19

**Authors:** Godwin A Ebughe, Ima-Obong Ekanem, Ayobele J Omotoso, Marcus Inyama, Thomas U Agan, B U Ago, A Ibangha, Denis Nkangha, U Etiuma, Grace Inah

**Affiliations:** 1 Department of Pathology, University of Calabar Teaching Hospital, Calabar, Nigeria; 2 Department of Haematology, University of Calabar Teaching Hospital, Calabar, Nigeria; 3 Department of Obstetrics and Gynaecology, University of Calabar Teaching Hospital, Calabar, Nigeria; 4 Department of Ophthalmology, University of Calabar Teaching Hospital, Calabar, Nigeria; 5 Department of Surgery, University of Calabar Teaching Hospital, Calabar, Nigeria; 6 Department of Radiology, University of Calabar Teaching Hospital, Calabar, Nigeria

## Background

Both AIDS defining malignancies and non AIDS defining malignancies occurring in HIV infected persons are poorly documented in Nigeria. Our hospital is a reference centre located in one of the South-South states, which has a HIV sero prevalence of 8% [1] (http://data.unaids.org/pub/Report/2010/nigeria_2010_country_progress_report_en.pdf), with a low antiretroviral coverage [1].

## Material and methods

A five year retrospective study was carried out to review the frequency of diagnosis of three tumours classified as AIDS defining malignancies (Kaposi sarcoma, non Hodgkin lymphoma, cervical cancer) and one non AIDS defining malignancy (squamous cell carcinoma of the conjunctiva), also commonly diagnosed in these patients. Records of the patients which are histologically confirmed and diagnosed between 1^st^ January 2005 and 31^st^ January 2009 were sorted out and their retroviral status classified.

## Results

A total of 4123 histologically confirmed biopsies were received, 852 (21%) were cancers, 24 (2.8%) were Kaposi sarcoma (KS), 8 (33%) KS occurred in females, range 21-60 years (y), 16 (67%) in males, range 19-60 y and 17 (71%) of KS were AIDS associated, 6 (35%) females and 11 (65%) males. Thirty five 35 (4.1%) of cancers were Non Hodgkin lymphomas including Burkitt’s lymphomas, 8 (23%) in females, range 6-60 y and males 27 (77%), range 6-71 y. Two 2 (5.7%) were AIDS associated 2 (100%) were males on long standing antiretroviral treatment. Cervical cancers accounted for 84 (9.9%) all cancers and 14 (17%) occurred in HIV positive patients age range. Conjunctival squamous cell carcinomas were 13 (1.5%) of all cancers, 6 (46%) females 7 (54%) males. Two 2 (15%) occurred in HIV positive patients.

## Conclusion

AIDS associated malignancies appear to be very common in this environment and perhaps non AIDS associated malignancies may be on the increase. Under-reporting and lack of capacity may account for the fewer numbers reported in this environment.
